# Protective effects of cordycepin pretreatment against liver ischemia/reperfusion injury in mice

**DOI:** 10.1002/iid3.792

**Published:** 2023-03-14

**Authors:** Yunxia Liu, Mingwei Sheng, Lili Jia, Min Zhu, Wenli Yu

**Affiliations:** ^1^ Department of Anesthesiology Tianjin First Central Hospital Tianjin China

**Keywords:** apoptosis, cordycepin, inflammation, ischemia/reperfusion, TLR4/NF‐κB signaling

## Abstract

**Introduction:**

Cordycepin has been reported to exhibit hepatic protective and anti‐inflammatory properties. Here, we investigated the role of cordycepin in ischemia/reperfusion (IR)‐induced liver injury in a mouse model.

**Methods:**

Mice were pretreated with cordycepin by gavage for 3 weeks, followed by the establishment of the IR modeling. Liver injury, Suzuki's histological grading, hepatic apoptosis, and inflammatory responses were evaluated by biochemical and pathological analysis.

**Results:**

It was found that Cordycepin pretreatment at 50 mg/kg for 3 weeks attenuated IR‐induced liver injury, as reflected by the significant decrease of the levels of aspartate aminotransferase, alanine transaminase, lactate dehydrogenase, and low‐density lipoprotein. Cordycepin pretreatment also reduced histopathological changes, attenuated hepatocyte apoptosis, inflammatory responses in the livers of IR mice. Mechanically, toll‐like receptor 4/nuclear factor kappa‐B signaling in liver tissues was inhibited by Cordycepin pretreatment.

**Conclusions:**

In conclusion, Cordycepin pretreatment protects IR‐induced liver injury, which demonstrates its potential for the treatment of IR in the liver.

## Graphical abstract

Cordycepin pretreatment reduced apoptosis and inflammation markedly in ischemia/reperfusion (IR) liver. Cordycepin protects and improves liver functions in IR mouse model.

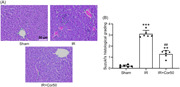

## 1 INTRODUCTION

Liver ischemia/reperfusion (IR) injury is common in liver surgery, especially in liver transplantation, hepatectomy, and trauma, which could cause high morbidity and mortality.^1^ Liver IR affects oxygen‐dependent cells firstly, after restoring blood flow and oxygen supply, reperfusion further aggravates the damage caused at the cellular level.^2^, ^3^ Despite great advances in diagnostic and surgical techniques, there is still lacking specific treatment to prevent IR‐induced liver injury.^4^, ^5^

Intracellular injury processes and inflammatory responses are two major characteristics of liver IR injury.^6^ Natural products such as resveratrol and cordycepin have been explored for their protective effects against liver injury.^7^ Cordycepin is an insect fungus derived active ingredient, which has been reported to exhibit antioxidant, antidepressant, antitumor, antiviral, and anti‐inflammatory activities. Cordycepin scavenges oxygen free radicals, improves antioxidant capacity, and attenuates age‐related oxidative stress.^8^, ^9^, ^10^, ^11^ Qing et al.^12^ reported that cordycepin activated the antioxidant defense response to prevent myocardial IR injury through upregulating heme oxygenase expression. In sepsis mouse model, cordycepin suppressed the lung and liver injury and immunodeficiency via induction of M1/M2 macrophage polarization.^13^ Moreover, cordycepin pretreatment effectively reduced the GaN/lipopolysaccharides (LPS)‐induced acute liver injury.^14^ In addition, cordycepin treatment protected the liver from inflammation and fibrosis through downregulation of lipid metabolism and inflammatory responses in the nonalcoholic fatty liver model.^15^

All these studies indicate that cordycepin possesses protective properties for both acute and chronic liver damages. However, the potential therapeutic effects of cordycepin in the context of IR‐induced liver injury remains obscure. In view of this, we intend to study the protective effects of cordycepin preconditioning on liver IR using a specific mouse hepatic ischemia‐reperfusion model.

## 2 MATERIALS AND METHODS

### 2.1 Animal study

A total number of 102 male C57BL/6 mice (18–22 g, 8–12‐week‐old) were ordered from GemPharmatech. Animal studies were performed in strict accordance with the NIH guidelines for the care and use of laboratory animals (8th edition, NIH), which were also approved by the Institutional Animal Care & Use Committee at Tianjin First Central Hospital (#2020N198KY). Mice were housed in pathogen‐free facility with 12‐hour light‐dark cycles. Three doses of cordycepin (5, 25, and 50 mg/kg/day, 99.2% purity, Shanghai Winherb Medical) were used for mouse pretreatment through daily oral gavage for consecutive 3 weeks. A partial liver IR model was established as previously described.^16^ Briefly, blood flow to the left lateral and median lobes of mice liver was blocked for 90 min to initiate liver reperfusion. Six hours after reperfusion, all mice were euthanized by CO_2_ inhalation for the following biochemical analyses.

### 2.2 Determination of liver function

The serum aspartate aminotransferase (AST), alanine transaminase (ALT), lactate dehydrogenase (LDH), and low‐density lipoprotein (LDL) were measured using corresponding kits provided by Stanbio Laboratory. SpectraMax M Microplate Readers (Molecular Devices) was used to perform the measurement according to the manufacturer's instructions.

### 2.3 Histology analysis

The liver tissues of the mice were fixed with 4% paraformaldehyde and then embedded in paraffin. Sections (5 μm) were cut and subject to hematoxylin and eosin (H&E) staining. Liver injury was evaluated based on Suzuki's histological grading.^17^

### 2.4 Apoptosis assay

Hepatocyte apoptosis was assessed using a TUNEL Apoptosis Assay Kit (Beyotime). After the 4′,6‐diamidino‐2‐phenylindole staining, the slides were visualized by using an sp5 Leica fluorescence microscope.

### 2.5 Pharmacokinetic assay

The in vivo pharmacokinetic profile of cordycepin was determined by following the previously reported protocol with minor modification.^18^ Mice were orally administered with cordycepin (50 mg/kg) and blood samples (200 µL) were collected at different time points (0.25–12 h after administration, *n* = 6). The metabolite of cordycepin, 3′‐deoxyinosine, was measured as described previously.^19^

### 2.6 Quantitative real‐time polymerase chain reaction (qRT‐PCR)

RNA from liver tissues (*n* = 8, each group) was extracted using a commercial kit from Thermo Fisher. For qPCR analysis, 2 μg of total RNA was reverse‐transcribed using MMLV‐RT (Thermo Fisher) followed by qPCR reactions using SYBR green. The relative expression levels of *Bax* and *Bcl‐2* were normalized to *GAPDH*.

### 2.7 Western blot analysis

Liver samples (*n* = 8, each group) were homogenized by using lysis buffer (150 mM NaCl, 5 mM NaF, 50 mM Tris, 25 mM β‐glycerol, 1 mM sodium orthovanadate, 1% Triton X‐100, 10% glycerol, and 1 mM dithiothreitol) supplemented with fresh protease inhibitors (Roche). Immunoblotting experiments were performed as previously described.^14^ For immunoblots, antibodies against toll‐like receptor 4 (TLR4, diluted at 1:1000, ab45104), p65 (diluted at 1:1000, ab16502), p‐p65 (diluted at 1:1000, ab86299), and GAPDH (diluted at 1:2000, ab22048) were purchased from Abcam. The primary antibodies of Bax (diluted at 1:1000, sc‐7480) and Bcl‐2 (diluted at 1:1000, sc‐7382) were obtained from Santa Cruz Biotechnology, Inc.

### 2.8 Immunohistochemistry (IHC) staining

Liver tissues (*n* = 6, each group) were fixed by paraformaldehyde (4%) and embedded in paraffin, and cut into 5‐μm‐thick sections. Sections were incubated with F4/80 primary antibody (1–200 dilution, Santa Cruz Biotechnology, sc‐26643) for 1 h at 37°C and then incubated in secondary antibody following three washes. Sections were colored using diaminobenzidine (DAB) kit and counterstained with hematoxylin.

### 2.9 ELISA

Inflammatory factors, including tumor necrosis factor (TNF)‐α, monocyte chemoattractant protein‐1 (MCP‐1), and interleukin (IL)‐10), of liver tissues (*n* = 8, each group) were detected by using Mouse TNF alpha ELISA Kit (ab208348, Abcam), MCP‐1 Mouse ELISA Kit (Thermo Fisher Scientific), and Mouse IL‐10 ELISA Kit (ab255729, Abcam), respectively, and following the manufacturer's instructions.

### 2.10 Statistical analysis

Statistical analyses in this study were carried out using GraphPad Prism8. Statistical differences were determined by using analysis of variance and appropriate followed Dunn's multiple comparisons test for comparisons of more than two groups.

## 3 RESULTS

### 3.1 Cordycepin pretreatment attenuated IR‐induced liver injury

**Figure 1:**
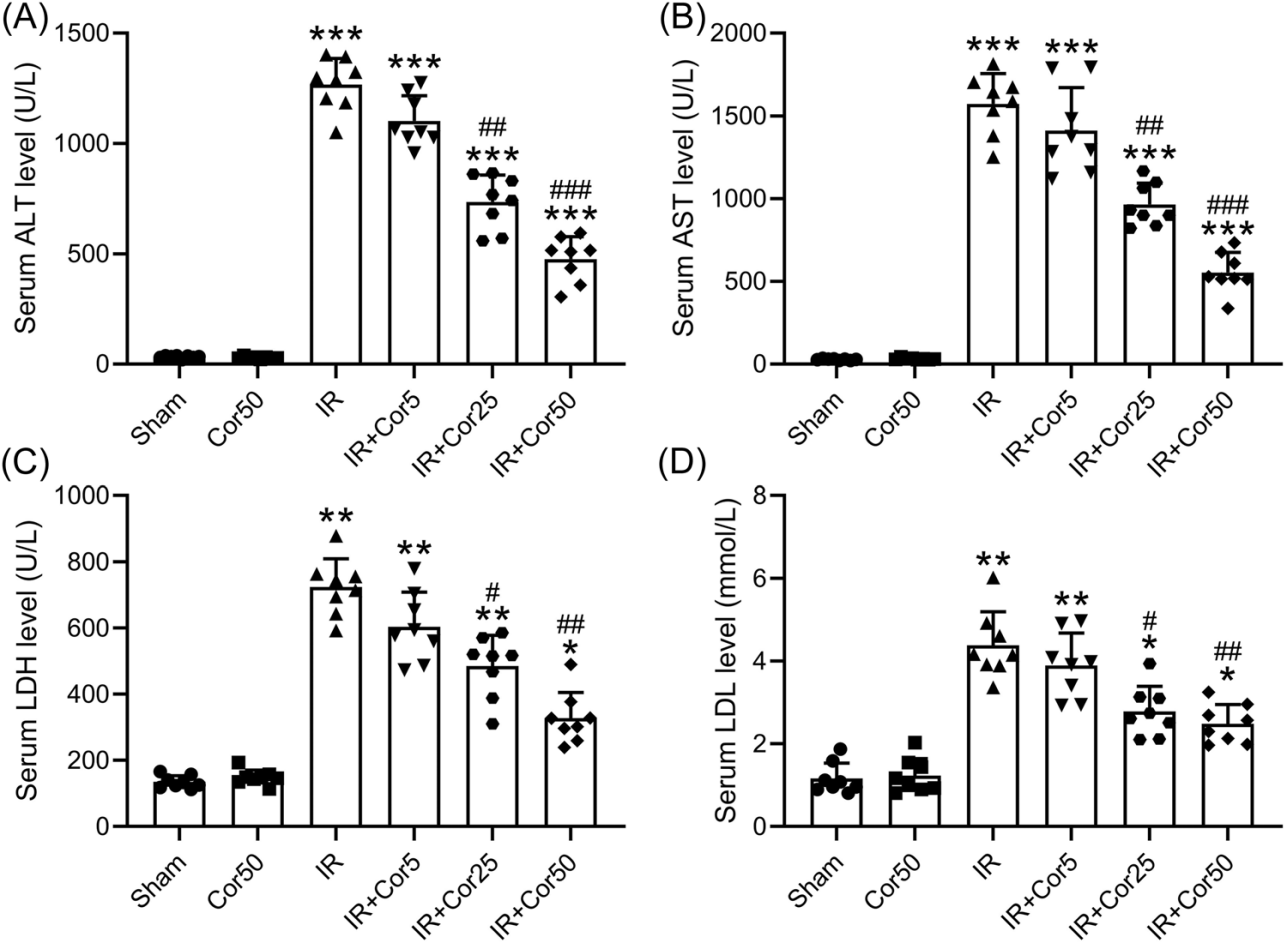
Cordycepin pretreatment attenuated IR‐induced liver injury in a dosage effect. Serum levels of ALT (A), AST (B), LDH (C), and LDL (D) among different groups. Data were shown as mean ± SD. Eight mice were used for each group. *p < .05, **p < .01, ***p < .001 compared with the sham group, ^#^ *p* < .05, ^##^ *p* < .01, ^###^ *p* < .001 compared with IR group. One‐way ANOVA followed Dunn's multiple comparisons test. ALT, alanine aminotransferase; ANOVA, analysis of variance; AST, aspartate aminotransferase; IR, ischemia/reperfusion; LDH, lactate dehydrogenase; LDL, low‐density lipoprotein; SD, standard deviation.

First, the IR‐induced liver injury was evaluated by measuring the concentration of liver injury markers (serum ALT, AST, LDH, and LDL) in Sham and IR mice with or without 3‐week Cordycepin administration. In comparison with Sham, IR increased serum ALT, AST, LDH, and LDL significantly (Figure 1A–D), suggesting that this IR model can induce liver injury successfully. Interestingly, upon Cordycepin administration, the serum levels of the above liver injury markers were significantly reduced with the increasing of Cordycepin doses (5, 25, and 50 mg/kg). Interestingly, all four liver injury markers exhibited comparable level in Sham mice with vehicle and 50 mg/kg Cordycepin administration (Figure 1A–D), indicating that Cordycepin is safe and has no side effects on normal mouse liver function. Hence, the 50 mg/kg dose was used in the following experiments.

### 3.2 Cordycepin pretreatment improved IR‐induced liver injury

**Figure 2:**
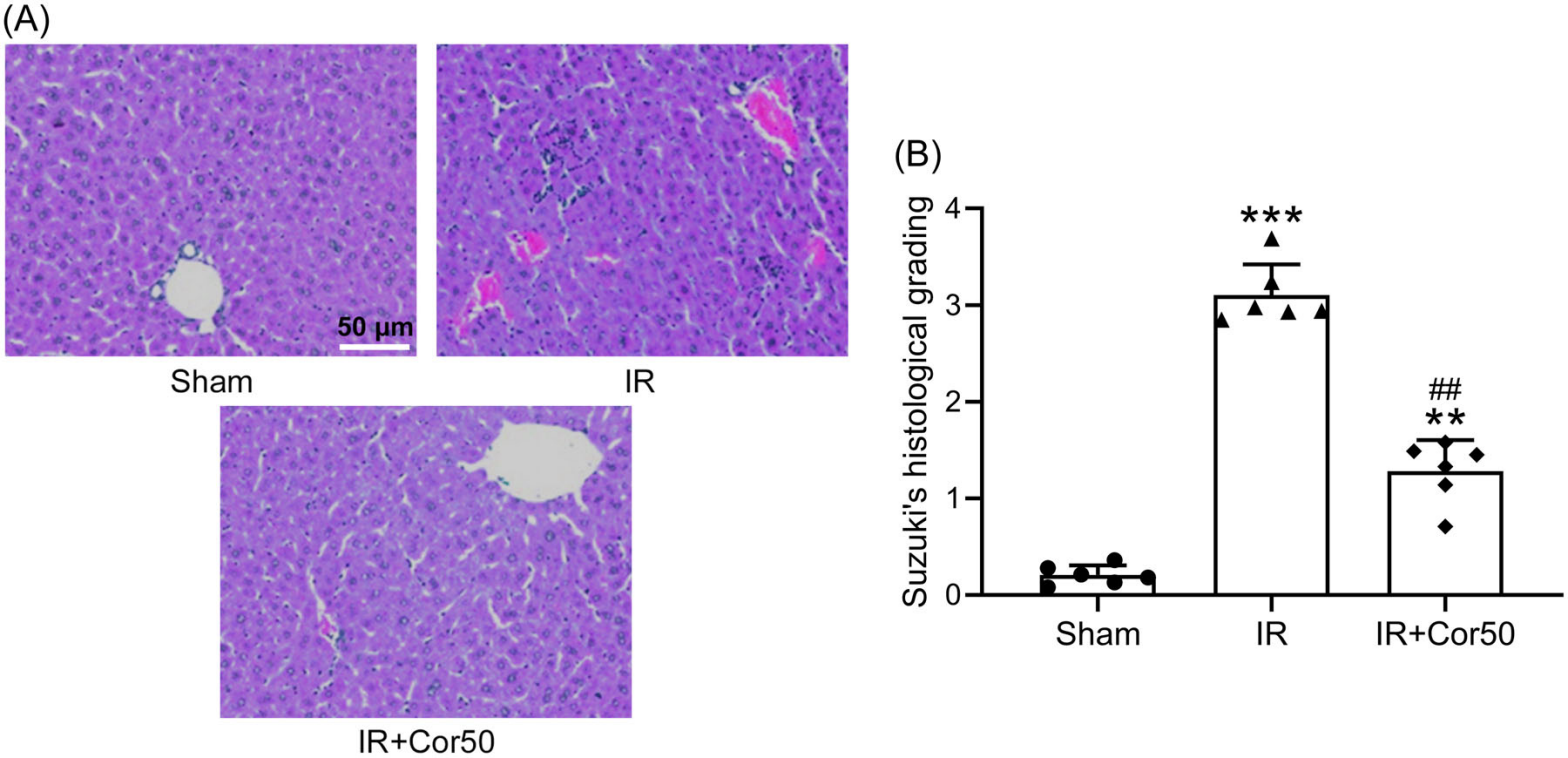
Effect of cordycepin pretreatment on histopathological changes of liver tissues after liver IR. (A) Representative H&E staining of liver tissues. (B) Suzuki's histological grading from the H&E staining. Data were shown as mean ± SD. Six mice were used for each group. The data point indicated the average score of one mouse from 8 field. **p < .01, ***p < .001 compared with the sham group, ^##^ *p* < .01 compared with the IR group. One‐way ANOVA followed Dunn's multiple comparisons test. ANOVA, analysis of variance; H&E, hematoxylin and eosin; IR, ischemia/reperfusion; SD, standard deviation.

To further evaluate IR‐induced liver injury, we performed H&E staining and quantized the injury by Suzuki's score. IR treatment significantly induced liver injury, and Suzuki's value of IR liver was elevated markedly compared to that in Sham liver (Figure 2A,B). However, upon 3 weeks cordycepin administration, Suzuki's score of IR + Cordycepin (IR + Cor50) was significantly deceased relative to that in IR liver (Figure 2B). Hepatic I/R injury results in increased infiltration of the immune cells, upon Cordycepin pretreatment, IR‐induced liver macrophage infiltration was significantly reduced compared to that in the IR group (Figure S1).

### 3.3 Cordycepin pretreatment inhibited IR‐induced hepatocyte apoptosis

**Figure 3:**
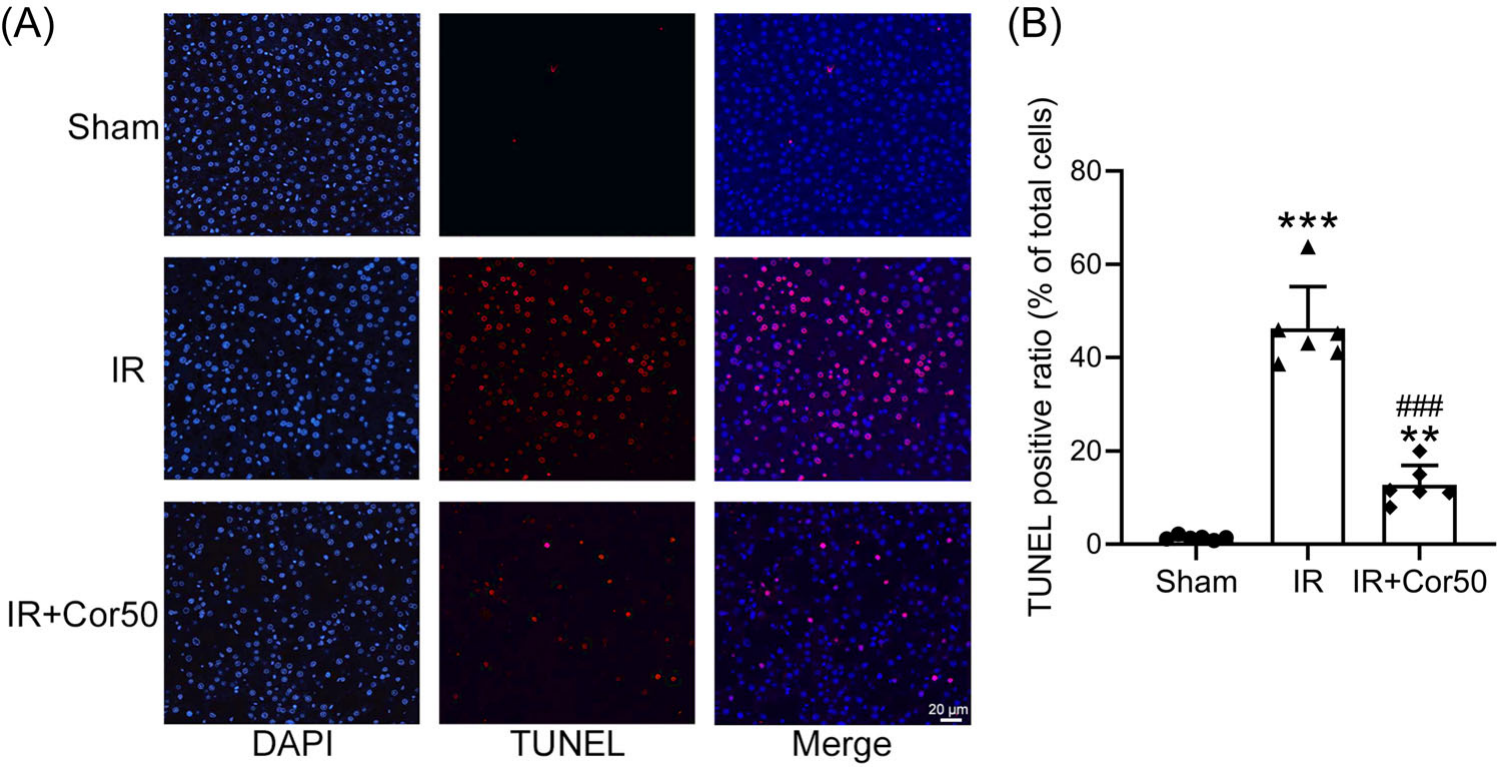
Cordycepin pretreatment attenuated liver IR‐induced hepatocyte apoptosis. (A) Representative figures of TUNEL assay among different groups. (B) Quantification of TUNEL positive ratio among different groups. Data were shown as mean ± SD. Six mice were used for each group. The data point indicated the average score of one mouse from eight fields. **p < .01, ***p < .001 compared with the sham group, ^###^ *p* < .001 compared wit the IR group. One‐way ANOVA followed Dunn's multiple comparisons test. ANOVA, analysis of variance; DAPI, 4′,6‐diamidino‐2‐phenylindole; IR, ischemia/reperfusion; SD, standard deviation; TUNEL, terminal deoxynucleotidyl transferase dUTP nick end labeling.

**Figure 4:**
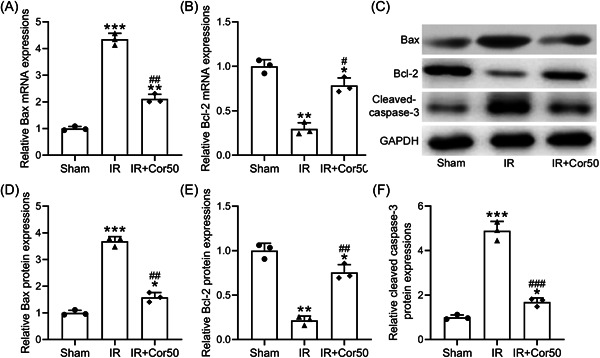
Cordycepin pretreatment attenuated liver IR‐induced hepatocyte apoptosis. qRT‐PCR was used to analyze the mRNA levels of Bax (A) and Bcl‐2 (B) in liver tissues among different groups. (C) Western blot analysis was used to measure the protein levels of Bax, Bcl‐2, and cleaved caspase‐3 in liver tissues. GAPDH was used as a loading control. The relative expressions were normalized to sham (D–F). Data were shown as mean ± SD. Eight mice were used for each group. The data were gotten from three repeated experiments using mixed homogenate in each group. *p < .05, **p < .01, ***p < .001 compared to sham group, ^#^ *p* < .05, ^##^ *p* < .01, ^###^ *p* < .001 compared with the IR group. One‐way ANOVA followed Dunn's multiple comparisons test. ANOVA, analysis of variance; IR, ischemia/reperfusion; mRNA, messenger RNA; qRT‐PCR, quantitative real‐time polymerase chain reaction; SD, standard deviation.

Hepatocyte apoptosis is one of the major features of liver injury, then we detected the apoptotic levels in the liver of control and cordycepin treated mice by TUNEL assay and marker gene expression analysis. TUNEL positive ratio in IR liver was increased markedly, suggesting lots of hepatocyte apoptosis occurred (Figure 3A,B). While TUNEL positive ratio was significantly reduced in the liver of IR mice with Cordycepin administration (Figure 3B). Bax is an apoptosis marker gene, the expression of which was significantly increased in IR liver and decreased in IR + Cor50 liver (Figure 4A). In contrast, the expression of apoptosis inhibitory protein, Bcl2, was decreased in IR liver and increased in the liver of Cordycepin treated mice (Figure 4B). Same expression pattern of Bax and Bcl2 was observed on the protein level (Figure 4C–E). Moreover, cleaved caspase‐3, another apoptosis marker, was increased in liver tissues of IR mice, while it was significantly decreased in liver tissues of mice with 3 weeks cordycepin pretreatment (Figure 4F). These data indicated that cordycepin preconditioning suppresses IR‐induced hepatocyte apoptosis.

### 3.4 Toll‐like receptor 4 (TLR4)/nuclear factor kappa‐b (NF‐Κb) signal pathway was involved in the protective effects of cordycepin

**Figure 5:**
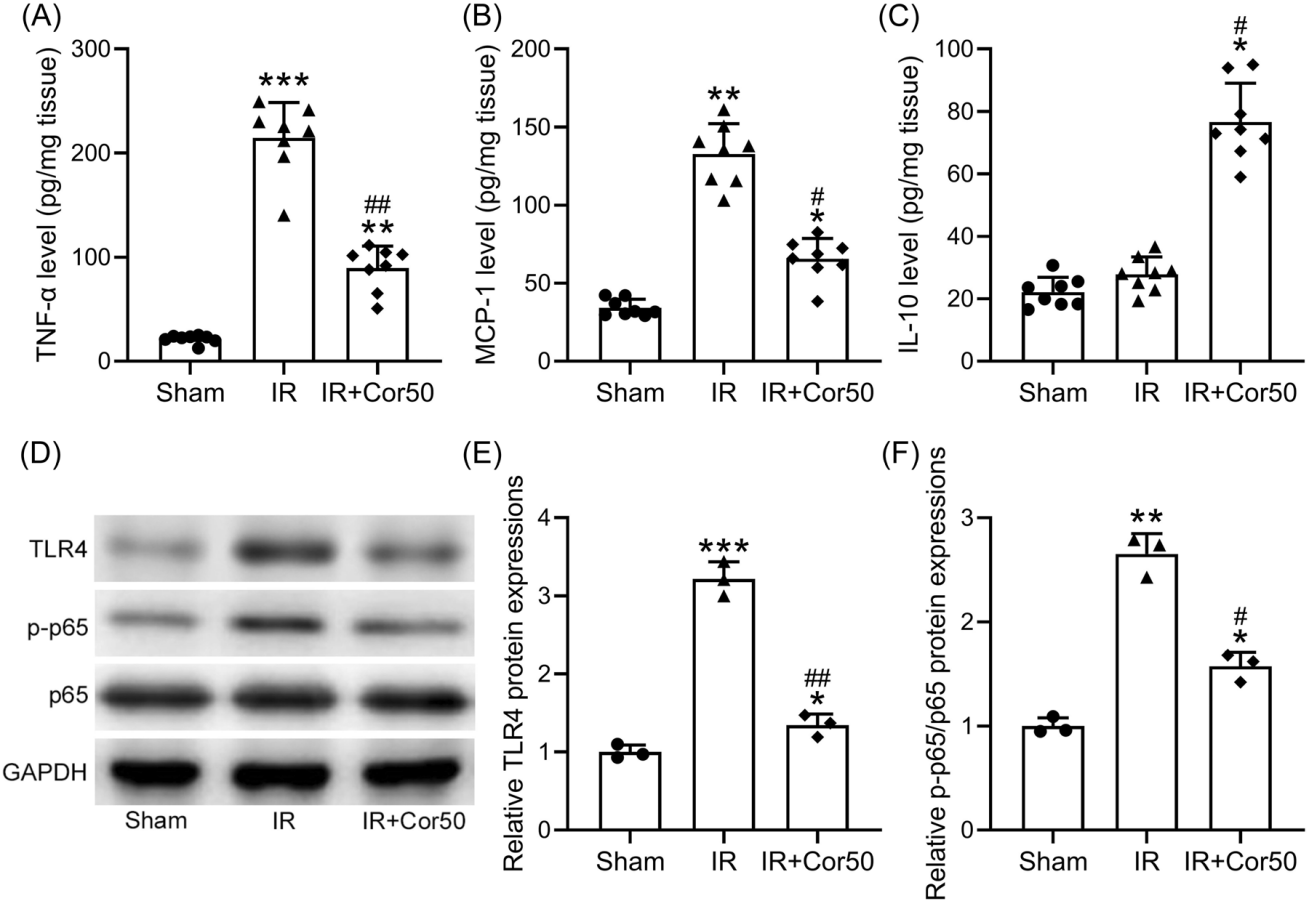
Cordycepin pretreatment inhibited TLR4/NF‐κB signal in liver tissues after liver IR. ELISA was used to measure the levels of TNF‐α (A), MCP‐1 (B), and IL‐10 (C) in liver tissues among different groups. (D) Western blot analysis was used to measure the protein levels of TLR4, p‐p65, and p65. GAPDH was used as loading control. The relative expressions were normalized to sham (E and F). Data were shown as mean ± SD. Eight mice were used for each group and *n* = 8 for ELISA analysis. The data were gotten from three repeated experiments using mixed homogenate in each group for western blot analysis. *p < .05, **p < .01, ***p < .001 compared with the sham group, ^#^ *p* < .05, ^##^ *p* < .01 compared with the IR group. One‐way ANOVA followed Dunn's multiple comparisons test. ANOVA, analysis of variance; ELISA, enzyme‐linked immunosorbent assay; IL, interleukin; MCP‐1, monocyte chemoattractant protein‐1; TNF‐α, tumor necrosis factor‐α.

Since TLR4/NF‐κB signal pathway plays a key role in hepatocyte apoptosis and liver injury,^20^ we further investigated whether this signaling pathway was involved in the protective effects of cordycepin against I/R injury. The levels of TNF‐α and MCP‐1, as two important inflammatory factors in the liver, were both significantly increased in liver tissues of mice with IR treatment (Figure 5A,B). While after 3 weeks of cordycepin pretreatment, TNF‐α and MCP‐1 levels were reduced markedly in the liver of mice (Figure 5A,B). IL‐10 has been reported to ameliorate liver injury by inhibiting proinflammatory responses, which is partly regulated through the inhibition of TLR4/NF‐κB signaling pathway.^21^, ^22^ It was further found that IL‐10 level was comparable in Sham and IR livers, however, cordycepin pretreatment significantly increased IL‐10 concentration in liver tissues (Figure 5C). Moreover, western blot results showed that TLR4 and p‐p65 were upregulated in IR liver, while both were decreased after 3 weeks cordycepin pretreatment (Figure 5D–F). Furthermore, Cordycepin pretreatment inhibited IκB phosphorylation in liver tissues after liver IR (Figure S2). These data suggested that Cordycepin pretreatment inhibited TLR4/NF‐κB signaling in IR liver, which might contribute the protective role of Cordycepin on IR‐induced liver injury.

In addition, when cordycepin was administered to mice orally, it was not detected in plasma at all, at any time point. However, the metabolite 3′‐deoxyinosine was found to be absorbed systemically instead, and the plasma concentration‐time profiles were depicted in Figure S3.

## 4 DISCUSSION

Liver IR injury is a complex process containing the initial ischemic injury and subsequent reperfusion injury mediated by inflammation responses, leading to high morbidity and mortality.^4^, ^23^ A growing number of studies have confirmed that cordycepin has protective effects in rodent models of oxidative stress, infection, inflammation, and apoptosis.^24^, ^25^, ^26^ We demonstrated the protective effect of cordycepin on IR‐induced liver injury. Our findings indicated that mice pretreated with Cordycepin for 3 weeks could significantly alleviate the IR‐induced liver injury, as evidenced by the decrease of LDH, LDL, ALT, and AST in serum, and the reduction of the Suzuki's histological grading. Mechanistically, cordycepin pretreatment ameliorated IR‐induced hepatocyte apoptosis and inflammatory response, in which the inhibition of the activation of TLR4/NF‐κB signaling pathway may be involved.

Apoptosis is one of the major features of IR‐induced liver injury.^27^ Apoptotic responses include inflammatory cells, soluble stimuli, resident parenchymal cells, and stellate cells, which is the first cellular response to many liver toxic events and accompanies viral hepatitis, nonalcoholic fatty liver disease, cholestasis, and IR injury.^28^, ^29^, ^30^ In this study, we found that IR treatment significantly increased the serum ALT, AST, LDH, and LDL levels, and more liver injury areas were observed in IR liver tissues reflecting by elevated Suzuki's histological grading. TUNEL assay revealed that IR liver had remarkably increased TUNEL positive ratio compared to that in control liver. Jiang et al.^16^ reported that Glycyrrhizin acid has anti‐inflammatory and hepatoprotective properties through suppressing cell apoptosis and infiltration of neutrophils. Our findings showed cordycepin can inhibit the protein levels of Bax and cleaved caspase‐3, two apoptotic markers, and increase the expression of Bcl2, an antiapoptotic protein, in IR liver. Moreover, I/R treatment caused many necrotic hepatocytes in centrilobular region, and cordycepin pretreatment can improve this phenomenon, indicating that cordycepin on the liver mainly acts on hepatocytes. Similarly, the protective effect of cordycepin on hepatocytes was observed in GalN/LPS‐induced liver injury mice.^14^ These results revealed that cordycepin attenuates IR‐induced hepatic injury by inhibiting apoptosis.

Liver IR stimulates the innate immune system to drive the full development of inflammatory hepatocyte injury.^31^ TNF‐α is one of major mediator of hepatotoxicity, for example, lipopolysaccharide could induce liver injury through upregulation of TNF‐α in obese mice.^32^, ^33^ Monocyte chemoattractant protein‐1 (MCP‐1) is a chemokine, which is significantly elevated in mouse models and patients with alcoholic liver disease.^34^ In line with the above findings, liver IR treatment increased the TNF‐α and MCP‐1 levels, while 3 weeks of cordycepin pretreatment could significantly reduce the production of both TNF‐α and MCP‐1 in IR liver. Studies demonstrated that hepatic injury resulted in increased infiltration of the immune cells.^35^, ^36^ Cordycepin pretreatment significantly reduced the IR‐induced liver macrophage infiltration, indicating liver microenvironment might be reprogrammed upon cordycepin pretreatment. Moreover, the expression of IL‐10, an anti‐inflammatory cytokine,^37^ was increased remarkably in liver tissues with Cordycepin pretreatment. Several studies have demonstrated the key role of TLR4/NF‐κB signaling pathway in the regulation of apoptosis and inflammatory response.^38^, ^39^ This study showed that cordycepin pretreatment inhibited TLR4/NF‐κB signal (reduction of TNF‐α, MCP‐1, TLR4, p‐p65, and p‐IκB) in liver after IR, recently Ding et al. reported the protective role of cordycepin in hepatic IR through MAPK/NF‐κB signaling pathway,^40^ the role of TLR4/NF‐κB signal and other pathways in cordycepin mediated hepatic IR improvement need to be further verified by using KO mouse lines and inhibitors in the future studies.

## 5 CONCLUSION

Our data highlights the potential of cordycepin pretreatment against IR‐induced liver injury in mice, which might be through the inhibition of TLR4/NF‐κB signaling pathway. The upstream effectors and detailed molecular mechanisms underlying the protective role of Cordycepin warrant further investigation in the future.

## AUTHOR CONTRIBUTIONS

**Yunxia Liu**: Conceptualization; data curation; writing—original draft. **Mingwei Sheng**: Data curation; formal analysis; writing—original draft. **Lili Jia**: Data curation; formal analysis; writing—original draft. **Min Zhu**: Data curation; formal analysis; writing—original draft. **Wenli Yu**: Conceptualization; data curation; formal analysis; writing—original draft.

## CONFLICT OF INTEREST STATEMENT

The authors declare no conflict of interest.

## ETHICS STATEMENT

The animal experiments in this study were performed following procedures approved by the Institutional Animal Care & Use Committee at Tianjin First Central Hospital. This study was performed in strict accordance with the NIH guidelines for the care and use of laboratory animals (8th edition, NIH).

## Supporting information

Supplementary information.

## Data Availability

The raw data supporting the conclusions of this article will be made available by the authors, without undue reservation.
