# Supplementary material for: Protective effects of cordycepin pretreatment against liver ischemia/reperfusion injury in mice
Source: Immun Inflamm Dis. 2023 Mar 14;11(3):e792. doi: 10.1002/iid3.792 (PMC10013135; doi:10.1002/iid3.792)
Supplement: Supplementary file 1 — Supplementary information. [file IID3-11-e792-s001.docx]

**Supplementary materials**

**
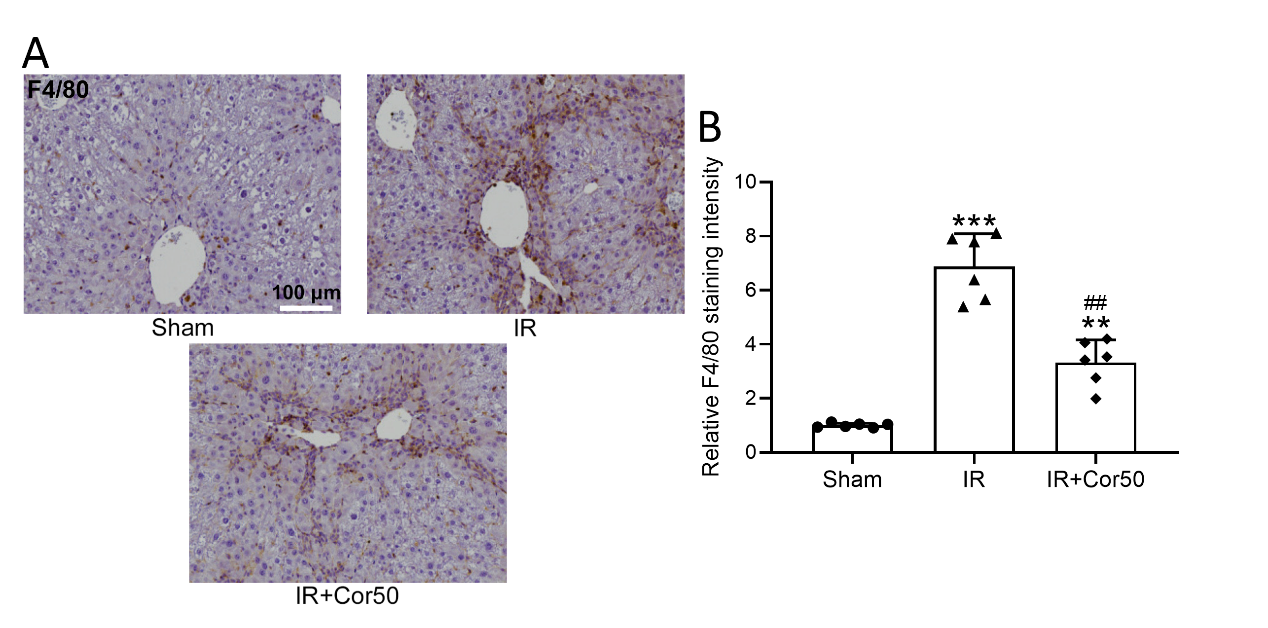
**

**Figure S1. Cordycepin pretreatment attenuated IR-induced liver macrophage infiltration.** A, representative F4/80 staining of liver tissues. B, relative staining intensity of F4/80 in liver. Data were shown as mean ± sd. 6 mice were used for each group. The data point indicated the average score of one mouse from 8 fields. **p < 0.01, ***p < 0.001 compared to sham group, ##p < 0.01compared to IR group. One-way ANOVA followed Dunn's multiple comparisons test.


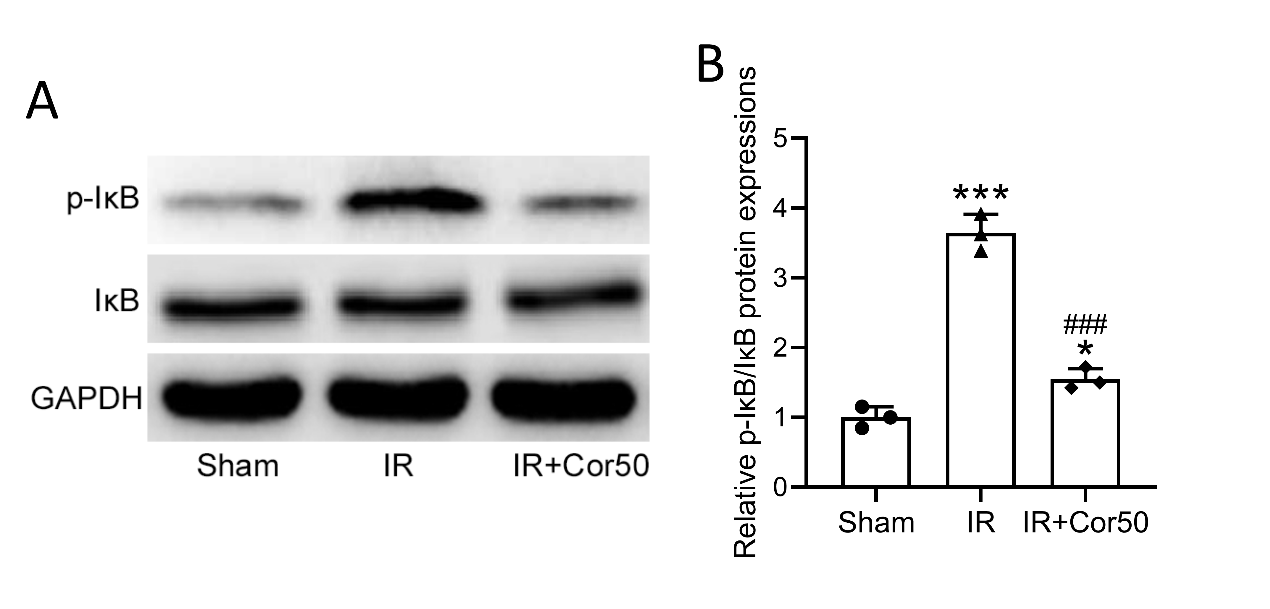


**Figure 2.** **Cordycepin pretreatment inhibited IκB phosphorylation in liver tissues after liver IR.** Western blotting was used to measure the protein levels of IκB and p-IκB (A). GAPDH was used as loading control. The relative expressions were normalized to sham (B). Data were shown as mean ± sd. 3 repeated experiments using mixed homogenate in each group for Western blotting. *p < 0.05, ***p < 0.001 compared to sham group, ###p < 0.001 compared to IR group. One-way ANOVA followed Dunn's multiple comparisons test.


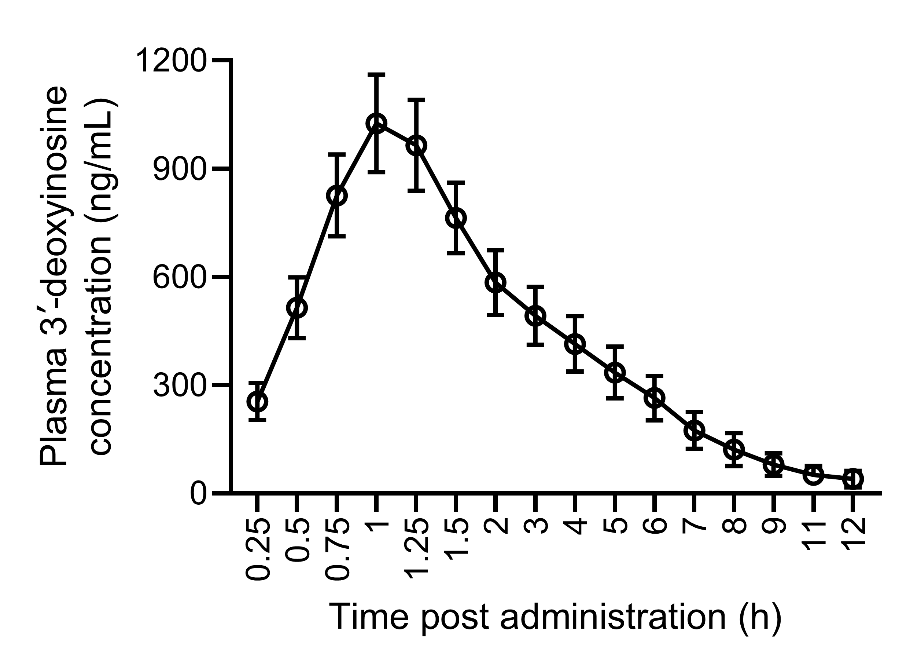


**Figure S3. In vivo pharmacokinetic profiles of cordycepin’s metabolite, 3’-deoxyinosine, following oral gavage administration of cordycepin in mice at 50 mg/kg (n = 6).**
